# Exploring the links between type and content of virtual background use during videoconferencing and videoconference fatigue

**DOI:** 10.3389/fpsyg.2024.1408481

**Published:** 2024-09-19

**Authors:** Benjamin J. Li, Heng Zhang

**Affiliations:** Wee Kim Wee School of Communication and Information, College of Humanities, Arts, and Social Sciences, Nanyang Technological University, Singapore, Singapore

**Keywords:** videoconferencing, videoconference fatigue, well-being, virtual backgrounds, LC4MP

## Abstract

The popularity of remote working in recent years has led to a rise in the use of videoconferencing tools. However, these communication tools have also given rise to a phenomenon known as videoconference fatigue (VF). Using the limited capacity model of motivated mediated message processing and impression management theory as the theoretical framework, this study explores how different types and content of virtual backgrounds in videoconferencing influence people’s VF and well-being. A survey of 610 users of videoconferencing tools revealed significant variations in the content and type of virtual backgrounds used during videoconferences. Our findings highlight three main points: first, there is a significant relationship between the use of virtual backgrounds and VF; second, pairwise comparisons showed that the type of virtual background significantly influences the amount of VF experienced by users; third, the content of virtual backgrounds also significantly impacts the level of VF experienced by users. These results suggest that careful selection of virtual backgrounds can mitigate VF and improve user well-being. Theoretical and practical implications are discussed.

## Introduction

Videoconferencing, a method of face-to-face telecommunication, has surged globally since the pandemic (Elbogen et al., 2022). Users of *Zoom* skyrocketed from 10 million to 300 million between December 2019 and April 2020 (Evans, 2020). Videoconferencing is now widely used in workplaces, education, and personal relationships (Mukan and Lavrysh, 2020; Gan, 2021; Nehe, 2021). While it enhances efficiency and productivity for remote work and allows students to continue virtual classes, it also leads to videoconference fatigue (VF). VF is defined as a “non-pathological tiredness arising from videoconferencing which manifests in physical, emotional, cognitive and social ways” (Li and Yee, 2023, p. 183). If left unchecked, VF can result in negative impact on users’ psychological and physical well-being (Queiroz et al., 2023), impair personal and social experiences (Nguyen et al., 2022; Fauville et al., 2023), diminished happiness in relationships (Bailenson, 2021), emotional exhaustion (Johnson and Mabry, 2022), psychological distress (Deniz et al., 2022), and life satisfaction (Deniz et al., 2022).

Research has identified factors contributing to videoconference fatigue (VF), such as mirror anxiety, social norms, and internet connection issues (Bailenson, 2021; Döring et al., 2022; Riedl, 2022; Li and Yee, 2023). Proposed measures to alleviate VF include reducing meeting duration, increasing breaks (Brown et al., 2020; Bailenson, 2021; Amponsah et al., 2022), turning off cameras (Wiederhold, 2020), discouraging multitasking (Döring et al., 2022), and improving equipment and connection quality (Nesher Shoshan and Wehrt, 2022). However, some of these solutions are beyond the control of the user. Companies and schools usually set meeting times and require users to keep their cameras on (Maimaiti et al., 2021; Rodeghero et al., 2021; Petchamé et al., 2022), while some users are pressured to multitask at home due to family responsibilities (Li et al., 2022).

Virtual backgrounds are a popular option in videoconferencing platforms, and users can choose the type of virtual background according to their preference (Cook et al., 2023). However, only a few studies have explored the effect of virtual backgrounds, with their focus mainly on how virtual backgrounds can positively influence people’s first impressions and creativity during videoconferencing (Hwang et al., 2021; Palanica and Fossat, 2022; Cook et al., 2023). With these findings in mind, there is reason to believe that virtual backgrounds can likely aid in alleviating the experience of VF. However, these studies only focus on user creativity at work, as well as the management and perception of first impressions, without addressing VF.

Previous research on computer-mediated communication (CMC) has shown that users often have more control over managing their image and selectively disclosing information (Joinson, 2001). This self-presentation behavior can make users more focused on their image and how they are perceived by others, rather than on the information and feelings of others. As a form of CMC, videoconferencing is significantly different from face-to-face communication. One of the most notable contrasts between video chats and face-to-face discussions is the presence of a self-view screen in video chats (Shin et al., 2023). In typical face-to-face conversations, people do not see their own facial expressions, gestures, or mannerisms. However, in video chats, the ability to monitor one’s own conversational behavior is provided by default (Shin et al., 2023). At the same time, previous studies have also pointed out that in video chats, users tend to focus more on themselves, even to the point of being unable to stop looking at themselves, and focus on self-related stimuli rather than other relevant stimuli (Alexopoulos et al., 2012; O’Gieblyn, 2021). Therefore, this study focuses on how users themselves use virtual backgrounds, rather than how they perceive other participants’ use of virtual backgrounds.

Overall, we use the limited capacity model of motivated mediated message processing (LC4MP, Lang, 2006) as the framework for this study in exploring the potential influence of virtual backgrounds on individuals during videoconferencing. The objectives of this study is to explore the relationships between virtual background use in videoconferencing and the experience of VF, and under what kind of mechanism. Also, we also explored the differences in the extent to which various types and contents of virtual backgrounds affect VF. Findings from this study should help researchers better understand the antecedent factors of VF and potentially alleviate its impact on users’ psychological and physical well-being.

## Literature review

### Virtual backgrounds in videoconferencing

Although there has been an increase in videoconference-related research, few studies have investigated people using virtual backgrounds during videoconferencing. According to existing research, three primary motivations drive users to utilize virtual backgrounds in videoconferencing. Firstly, many individuals use virtual backgrounds to hide their physical surroundings, addressing concerns about privacy and security. This use provides a sense of being in a controlled and secure physical environment (Vidolov, 2022). Secondly, virtual backgrounds enhance first impressions and demonstrate professionalism during important meetings (Hwang et al., 2021; Karabulut et al., 2022). They act as an extension of the professional image, allowing users to present a more idealized version of themselves (Douglas and McGarty, 2001). For instance, virtual office backgrounds help remote workers feel more professional (Karabulut et al., 2022). Thirdly, users may adopt virtual backgrounds to share a common setting with their communication partners, enhancing social presence and reducing distractions in settings like online classes (Aknuranda et al., 2021; Goethe et al., 2022).

Moreover, previous studies highlight the diversity in virtual backgrounds used in videoconferencing, including blurred, video, and still images with themes like nature, office settings, urban landscapes, and humorous images (Hwang et al., 2021; Cook et al., 2023). Users select these backgrounds for various reasons but face concerns about their impact. For instance, backgrounds themed with plants and books are rated highly for trustworthiness and competence, while home-themed backgrounds score lowest (Cook et al., 2023). Natural-themed backgrounds can enhance creativity compared to blurry or urban ones (Palanica and Fossat, 2022). Hwang et al. (2021) found that people choose office-related backgrounds for professional settings, funny images for familiar interactions, and nature or public spaces for unfamiliar ones. Karabulut et al. (2022) noted that employees prefer nonrevealing backgrounds to appear competent but may misalign with customers’ preferences for approachable and personable backgrounds. But these studies did not investigate the relationship between virtual background content and VF.

While some studies on videoconferencing mention virtual backgrounds, in-depth research and theoretical support are limited. Hwang et al. (2021) used ecological perception theory (EPT) and perception sensation theory (PST) to explain how sensory systems process environmental information, suggesting that virtual backgrounds can influence cognitive evaluations and user assessments. Palanica and Fossat (2022) employed attention restoration theory (ART) and stress reduction theory (SRT) to show that nature-themed backgrounds can restore cognitive resources and reduce stress, enhancing creativity. Cook et al. (2023) used the stereotype content model (SCM) to demonstrate that backgrounds with plants and books increase perceptions of trustworthiness and competence. Despite these theoretical frameworks, understanding virtual background use remains insufficient. Therefore, applying the limited capacity model of motivated mediated message processing (LC4MP) by Lang (2017) and impression management theory (IMT) by Goffman (1959), can provide further insights into how different background types and contents may impact cognitive resource consumption and fatigue, guiding more effective background selection.

### The limited capacity model of motivated mediated message processing (LC4MP)

Increased cognitive load can lead to VF manifesting in users experiencing reduced work efficiency and difficulty concentrating due to the need to process a large amount of information. As LC4MP was proposed in an attempt to understand the real-time dynamic interactions between individuals’ cognitive processing systems and media information, it can be extended to comprehend how users process information during CMC (Lang, 2006, 2017). When individuals use virtual backgrounds in videoconferencing, they replace the actual physical environment with a virtual one, altering the information received about the videoconferencing environment. When users utilize virtual backgrounds, their virtual background in the videoconferencing remains unchanged. Any new objects or people appearing around the user will not alter the information in the videoconferencing environment. This consistency helps maintain a stable and undisturbed virtual setting. Overall, users will not receive new information caused by changes in their physical environment during the videoconferencing.

LC4MP posits that individuals’ information processing capacity is limited (Shiffrin and Schneider, 1977; Basil, 1994; Lang, 2006, 2017). The human brain can only process a small portion of the information in the environment at a given time (Lang, 2006; Fisher et al., 2018). Previous research indicates that for users who do not use virtual backgrounds during videoconferencing, unexpected occurrences (such as other individuals appearing on camera while working from home) can introduce unintended new information (Hwang et al., 2021). According to LC4MP, when faced with new information, the human brain naturally exhibits an orienting response (OR) (Lang, 2006). Consequently, this may lead to insufficient information processing capacity, thereby affecting the handling of primary tasks during the videoconferencing. When users cannot efficiently process the primary tasks in a videoconferencing, they may experience decreased work efficiency and increased VF (Li and Yee, 2023, p. 183). As we know, not everyone has the luxury of having a dedicated room for work at home. Many people work in their living rooms, which may result in other family members entering the camera frame during videoconferencing. Additionally, when people work in places like train stations or coffee shops, they cannot prevent other people or objects from appearing in the background and thus in the videoconferencing. By using virtual backgrounds, they can avoid these situations, thereby preventing new information from appearing in the videoconferencing that might distract participants, consume cognitive resources, and contribute to VF. Therefore, using virtual backgrounds can prevent new information from disrupting the videoconferencing compared to not using virtual backgrounds. Based on this, we propose the following hypothesis:

*H1:* Users of virtual backgrounds during videoconferencing will experience less videoconference fatigue than those who do not use virtual backgrounds during videoconferencing.

Additionally, we understand that there are various types of virtual backgrounds, including image, video, and blurred backgrounds. These different types of virtual backgrounds introduce varying levels of new information to users. Blurred backgrounds likely generate the least amount of new information, followed by image backgrounds, which primarily introduce new information at the beginning of the videoconferencing. Video backgrounds refer to backgrounds made up of moving visual images rather than a single static image. In *Zoom*, an example of a default video background is a ten-second clip featuring the beach, where the palm trees are swaying and the waves are crashing on the shore. The clip then repeats itself through the videoconference as a virtual background. While it started out as a feature in *Zoom*, video backgrounds are now increasingly an additional option on various videoconference platforms such as *WebEx*. As video backgrounds generate more information as compared to static images or blurred backgrounds, we propose the following hypothesis:

*H2:* Users of video virtual backgrounds during videoconferencing will experience higher videoconference fatigue than those with image and blurred virtual backgrounds during videoconferencing.

Meanwhile, it is evident that the amount of new information generated by these different types of virtual backgrounds varies, leading to different cognitive demands on users. According to LC4MP, individuals have limited cognitive resources, and an overload of information can deplete these resources, resulting in cognitive load (Lang, 2006, 2017). In other words, cognitive load may arise from an imbalance between an individual’s cognitive capacity and the demands of processing visual and verbal information (Li and Yee, 2023). When users’ cognitive processing capacity is overwhelmed, videoconferencing participants are more prone to making errors in their work. Moreover, numerous studies have already established that cognitive load is one of the primary causes of VF (Shklarski et al., 2021; Nesher Shoshan and Wehrt, 2022; Li and Yee, 2023, p. 183). Therefore, we hypothesize that different types of virtual backgrounds may lead to varying levels of cognitive load, which in turn could result in different levels of VF. Based on this, we propose the following hypothesis:

*H3:* Users of video virtual backgrounds during videoconferencing will experience higher cognitive load than those with image and blurred virtual backgrounds during videoconferencing.

*H4a,b,c:* Among users of virtual backgrounds, the relationship between an individual’s use of (a) an image background, (b) a video background, and (c) a blurred background during videoconferencing and videoconference fatigue will be mediated by cognitive load.

### Impression management theory

When users select images or video backgrounds in videoconferencing, they can choose different types of content for these backgrounds. These varied virtual backgrounds can influence the first impressions participants have of the user. This phenomenon can be explained by impression management theory (IMT) (Goffman, 1959), which is often considered synonymous with self-presentation theory (SPT) (Nichols, 2020). IMT posits that humans are inherently actors, striving to present a certain image to others, and this leads to conscious or unconscious attempts to achieve a desired public image (Goffman, 1959; Nichols, 2020).

Previous research on virtual backgrounds has shown that users select different backgrounds to present a better version of themselves, aiming to appear more professional and leave a positive impression (Hwang et al., 2021; Cook et al., 2023). This demonstrates that people may use virtual backgrounds as a form of impression management. Goffman (1959) proposed that an individual’s self-presentation is a process of continuous and complex negotiation between two positions, each of which may involve multiple presentation strategies. An individual attempts to manage these presentation strategies through a continuous process of interpreting the audience, goals, and context. Therefore, users may choose different virtual background content for impression management based on different audiences or purposes.

According to previous research by Hwang et al. (2021), the content of virtual backgrounds can be broadly categorized into the following types: abstract, interior spaces, nature, public spaces, workplaces, funny, and others. Previous research on virtual backgrounds has indicated that users employ professional virtual background content (e.g., interior spaces, workplaces) to simulate an office setting, thereby making themselves appear more professional (Hwang et al., 2021; Cook et al., 2023). However, in professional environments, individuals may attempt to suppress personal values to align with organizational values (Hewlin et al., 2016), and these attempts at impression management can lead to fatigue (Al-Shatti et al., 2022).

In addition to professional virtual backgrounds, nature-based backgrounds have been recommended for impression management, as they can convey a sense of professionalism and yield positive outcomes for users (Ohly et al., 2016; Powers, 2020; Hwang et al., 2021). Yang et al. (2023) found that the use of nature-based virtual backgrounds resulted in higher perceived credibility compared to blank virtual backgrounds. Kelly et al. (2023) demonstrated that when teachers use nature-based backgrounds that reflect personal characteristics, it led to higher perceived cognitive learning among female students. Other studies reveal the benefits of using nature-based backgrounds, including alleviating stress and improving moods (Ohly et al., 2016), and lower fatigue and stress levels (Konishi et al., 2023).

Overall, whether using professional backgrounds (e.g., interior spaces, workplaces) or nature-based backgrounds, users can effectively engage in impression management and convey their desired image to others. However, compared to other types of backgrounds, nature-based backgrounds may help users feel more relaxed, as they do not impose the additional social pressures associated with professional backgrounds, such as suppressing personal values. Thus, we hypothesize that users who choose natural virtual backgrounds may experience less VF compared to those who use other types of virtual backgrounds, such as public spaces. To our knowledge, no study has investigated whether different types of virtual background content lead to varying levels of VF. To address this research gap, we propose the following research hypothesis:

*H5:* Users of nature-based virtual backgrounds during videoconferencing will experience less videoconference fatigue than those using other virtual backgrounds of other content types during videoconferencing.

## Method

### Data collection, sample and procedure

A nationwide online survey was conducted in Singapore in Spring 2023 with 610 participants. A data collection company was tasked to recruit participants who are current users of videoconferencing tools. Only those who met this requirement were allowed to complete the questionnaire. Participants were asked to think about their videoconferencing experiences generally when answering the questions. This was conveyed to them at the start of the questionnaire, and at the start of every section. Then, participants were required to select the type of background they use most frequently from the following options: image background, blurred background, video background, or no virtual background. Additionally, regarding the use of virtual backgrounds, when users chose to use an image or video background, they were further asked about the content of their virtual backgrounds. For the content of virtual backgrounds, participants were asked to select the type they use most frequently.

Respondents comprised 284 (46.6%) men and 326 (53.4%) women residing in Singapore. Participants were at least 21 years of age, and respondents ranged from 22 to 76 years old (*M_age_* = 43.68, *SD* = 12.25). They indicated that they worked from home for around 3 days per week (*M* = 3.09, *SD* = 1.95) and about 8 h per day (*M* = 7.61, *SD* = 2.25).

The study was approved by the Institutional Review Board of [Nanyang Technological University]. Participation was completely voluntary. Respondents provided their informed consent prior to data collection.

### Measures

#### Format of virtual background

To determine whether or not participants use virtual backgrounds during videoconference, as well as the format of virtual backgrounds they apply, participants were given four options to select from: “image”, “video”, “blurred background”, or “I do not use virtual backgrounds”.

#### Content of virtual backgrounds

According to Hwang et al. (2021), the most popular content of virtual backgrounds for videoconferences are: abstract, interior, nature, public spaces, workplaces, and funny. Thus, the participants were asked “what kind of virtual background setting do you usually use?” Since the aforementioned options may not provide a complete picture of the user’s usage, participants were allowed to fill in other types of virtual background content if the ones they use were not in the list.

#### Videoconference fatigue

VF was measured using 15 items. The items were adapted from the *Zoom Exhaustion & Fatigue Scale* (Fauville et al., 2021), which were rated on a five-point Likert scale (1: Not at all; 5: Extremely) and averaged to form an overall score for videoconference fatigue with strong reliability (*M* = 2.72, *SD* = 0.99, *α* = 0.97). The items contain five dimensions: general, visual, social, motivational, and emotional. General fatigue (*M* = 2.96, *SD* = 1.04, *α* = 0.93) comprised of three items (e.g., “How tired do you feel after videoconferencing?”). Visual fatigue (*M* = 2.49, *SD* = 1.14, *α* = 0.93) was measured with three items (e.g., “How blurred does your vision get after videoconferencing?”). Social fatigue (*M* = 2.78, *SD* = 1.14, *α* = 0.91) comprised of three items (e.g., “How much do you tend to avoid social situations after videoconferencing?”). Motivational fatigue (*M* = 2.89, *SD* = 1.12, *α* = 0.89) was measured with three items (e.g., “How much do you dread having to do things after videoconferencing?”). Emotional fatigue (*M* = 2.50, *SD* = 1.15, *α* = 0.92) comprised of three items (e.g., “How emotionally drained do you feel after videoconferencing?”).

#### Cognitive load

In this study, we primarily focus on the impact of information on users’ cognitive load. Thus, we adopted three items (information overload) from Lee et al. (2016) and rephrased them to fit the context of videoconferencing (α = 0.83, *M* = 3.34, *SD* = 0.89) (e.g., “I am often distracted by the excessive amount of information in videoconferencing.”). The questions were measured on a 5-point Likert scale item (1 = not at all, 5 = extremely).

### Data analysis

We conducted a confirmatory factor analysis (CFA) using JASP (Love et al., 2019). Descriptive statistics, hierarchical linear analysis, and the Kruskal-Wallis test were performed using *SPSS 29* IBM 2023, SPSS statistics (Version 29). Finally, we conducted a mediation model analysis using the PROCESS macro in SPSS (Hayes, 2013).

## Results

### Confirmatory factor analysis

We first utilized JASP (Love et al., 2019) to conduct a confirmatory factor analysis for the ZEF to demonstrate the factorial validity of the scale on the Singaporean population. In this model, a total of five factors were included. It indicates that items 1–3 corresponded to one factor; items 4–6 corresponded to a second factor; items 7–9 corresponded to a third factor; items 10–12 corresponded to a fourth factor; and items 13–15 corresponded to a fifth factor (see Table 1). The model showed a very good fit with chi-square (*p* < 0.001), CFI = 1, RESEA = 0.04, SRMR = 0.02. Acceptable cut-off values for CFI and TLI are 0.95 and greater (Cangur and Ercan, 2015). Values less or equal to 0.05 are considered to be a good fit for the RMSEA, and SRMR values less than 0.08 indicate a good fit (Hu and Bentler, 1999).

**Table 1:** Factor loadings.

Factor	Indicator	Coefficient	Standard error	z-value	*p*-value	95% CI
Factor 1	GENFAT 1	0.919	0.006	142.259	<0.001	0.906, 0.932
GENFAT	GENFAT 2	0.944	0.006	151.639	<0.001	0.931, 0.859
	GENFAT 3	0.946	0.007	139.997	<0.001	0.932, 0.959
Factor 2	VISFAT 1	0.895	0.007	129.040	<0.001	0.881, 0.908
VISFAT	VISFAT 2	0.963	0.006	152.784	<0.001	0.951, 0.976
	VISFAT 3	0.947	0.006	149.197	<0.001	0.935, 0.960
Factor 3	SOCFAT 1	0.913	0.008	108.509	<0.001	0.897, 0.930
SOCFAT	SOCFAT 2	0.890	0.007	121.615	<0.001	0.875, 0.904
	SOCFAT 3	0.938	0.008	123.990	<0.001	0.923, 0.953
Factor 4	MOTFAT 1	0.888	0.008	113.798	<0.001	0.873, 0.904
MOTFAT	MOTFAT 2	0.840	0.008	111.454	<0.001	0.825, 0.855
	MOTFAT 3	0.935	0.008	123.811	<0.001	0.920, 0.950
Factor 5	EMOFAT 1	0.931	0.006	143.933	<0.001	0.918, 0.943
EMOFAT	EMOFAT 2	0.933	0.006	152.723	<0.001	0.921, 0.945
	EMOFAT 3	0.914	0.006	144.278	<0.001	0.902, 0.926

### Descriptive statistics

Descriptive statistics were first conducted to gain an understanding of the prevalence and variety of virtual background use during videoconferencing. This study asked participants what form of virtual background they would use during videoconference. Respondents were given the options “image,” “video,” “blurred background,” and “I do not use virtual backgrounds.” There were 223 (36.6%) participants who selected “image,” 58 (9.5%) who chose “video,” 162 (26.6%) who chose “blurred background,” and 167 (27.4%) who selected “I do not use virtual backgrounds.” Participants who selected “image” and “video” were asked additional questions regarding the content of their virtual background. According to the results, 24 (8.5%) individuals selected “abstract,” 49 (17.4%) “interior spaces,” 140 (49.8%) “nature,” 15 (5.3%) “public spaces,” 41(14.6%) “workplaces,” 9 (3.2%) “funny,” and 3 (1.1%) “others.”

### Hierarchical linear analysis

Hierarchical linear regression was then used to determine the relationship between the use of virtual backgrounds and the VF of participants (H1). A model with demographic factors constituted the baseline mode, with virtual background use added as a binary factor (yes/no, yes = 1, no = 0) in the hypothesized model. Participants who chose “image,” “video” and “blurred background” were coded as *yes*, while those who selected “I do not use virtual background” were coded as *no*. Results of the baseline model showed that including the demographic variables (age, sex, education, and ethnicity) in Block 1 made a significant contribution to the regression model, *F* (4, 605) = 12.96, *p* < 0.001, and accounted for 7.9% of the total variation in VF of participants as compared to a model without any demographic factors. Adding virtual background use in Block 2 created a model that accounted for 9.5% of the total variation in VF of participants, *F* = (5, 604) = 12.66, *p* < 0.01. There was a 1.6% increase in *R*^2^ from the baseline to the hypothesized model, and this increase was significant. *F* (1, 604) = 10.61, *p* < 0.001. The findings suggest that the use of virtual background (image/video/blurred) is significantly related to higher VF.

In Block 1, the demographic variables were used as control variables. Age (*β* = −0.02, *p* < 0.001) was found to be significantly associated with participants’ VF, while gender (*β* = −0.12, *p =* 0.14), education (*β* = −0.01, *p* = 0.79), and ethnicity (*β* = −0.01, *p* = 0.81) were not. When an additional variable was added in the second stage, age (*β* = −0.02, *p* < 0.001) remained significantly associated with participants’ VF, while gender (*β* = −0.10, *p* = 0.21), education (*β* = −0.02, *p* = 0.53), and ethnicity (*β* = −0.01, *p* = 0.80) were not. Use of virtual background (image/video/blurred) is significantly related to higher VF (*β* = 0.29, *p* = 0.001) (see Table 2). H1 posits that participants who use virtual backgrounds will experience less VF than people who do not use virtual backgrounds. Taken together, H1 was not supported because the conclusion is contrary to our hypothesis.

**Table 2:** Use virtual background effect on VF.

	Model 1			Model 2		
Variable	B	SE B	β	B	SE B	β
Age (in years)	−0.023	0.003	−0.284***	−0.021	0.003	−0.262***
Sex	−0.116	0.079	−0.058	−0.099	0.078	−0.050
Education	−0.009	0.034	−0.010	−0.021	0.034	−0.025
Ethnicity	−0.013	0.056	−0.009	−0.014	0.056	−0.010
Use of virtual background				0.289	0.089	0.130**
R^2^	0.079			0.095		
Adj. R^2^	0.073			0.087		

B, Unstandardized; SE B, Coefficients; β, Standardized Coefficients.

Dependent Variable: Videoconference fatigue (VF).

*p < 0.05. **p < 0.01. ***p < 0.001.

### Kruskal-Wallis test

Due to the differences in sample size per group, the *SD* differences could affect results for both H2 and H5. Thus, the Kruskal-Wallis test was used to show that the normality assumptions of one-way ANOVA are not violated. Results revealed a statistically significant difference in VF across the different formats of virtual background (image, video, blurred and do not use) (H2), *X^2^* (*df* = 2, *p* < 0.001) =22.54.

Multiple comparisons revealed that the mean value of VF differed significantly between groups. Firstly, between the image (*M_VF_ =* 2.65, *SD* = 0.98) and video (*M_VF_ =* 3.44, *SD* = 1.03) background groups, and between the image and blurred (*M_VF_ =* 2.85, *SD* = 0.90) background groups, the mean value of VF differed significantly, with image being significant lower than video (*p* < 0.001) and blurred backgrounds (*p* = 0.041). Lastly, between blurred and video background groups, those who used a blurred background reported lower VF than those who used a video virtual background (*p* = 0.001) (see Table 3). These results provided answers to H2. More specifically, users with video backgrounds experienced higher VF than those using image and blurred virtual backgrounds during videoconferencing.

**Table 3:** Multiple comparison plot: type of virtual backgrounds on VF.

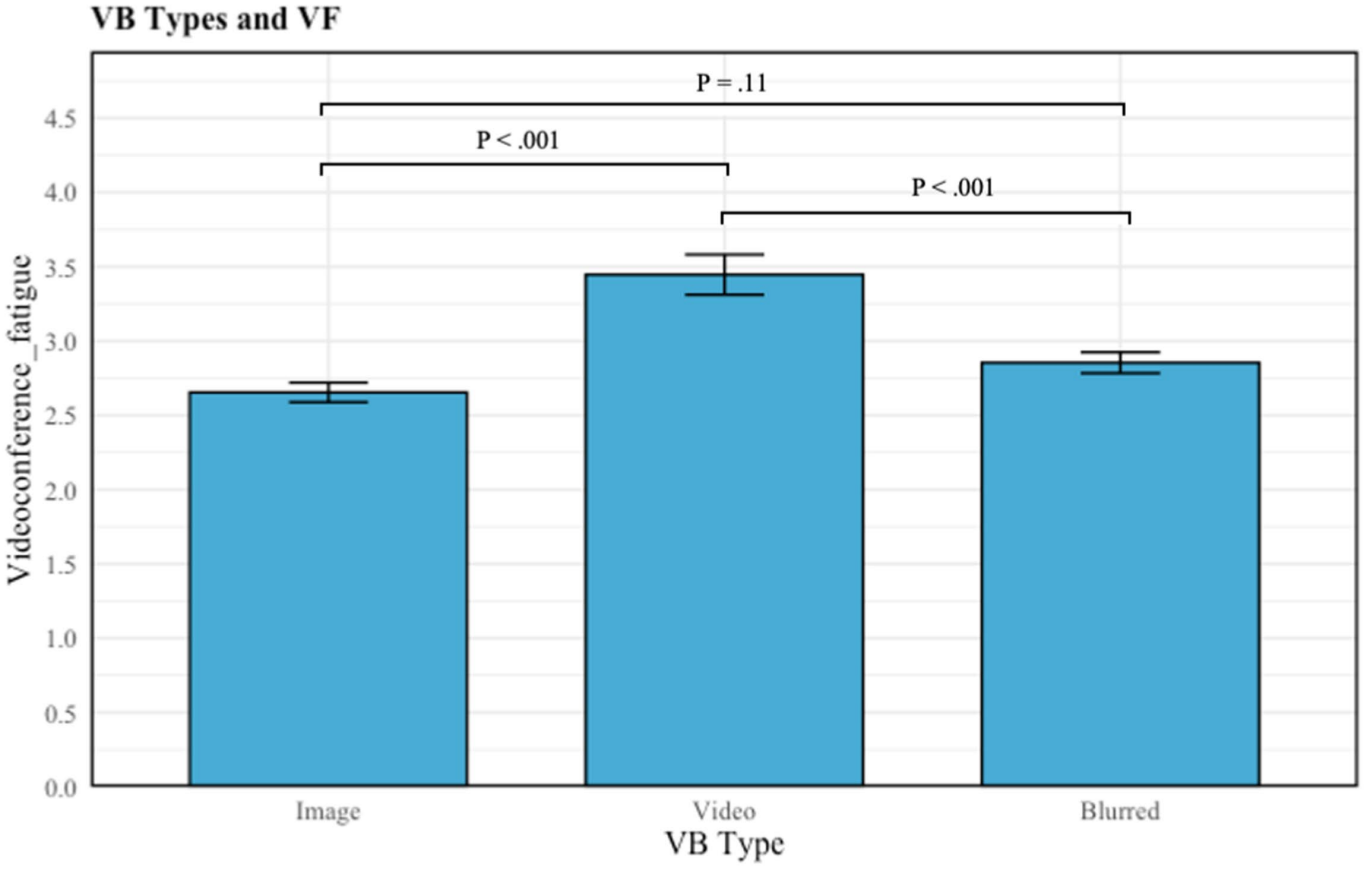

The Kruskal-Wallis Test was conducted to examine the differences in VF according to the different contents of the virtual background (H5). Significant differences were found among the seven categories of content (abstract, interior spaces, nature, public spaces, workplaces, funny, and others) (*X^2^* (*df* = 6, *p* = 0.023) = 14.63). In this study, the mean value of VF based on different content virtual backgrounds differs accordingly. The mean value of VF in each of the content categories are shown in Table 4. Multiple comparisons revealed that the mean value of VF differed significantly between some groups.

**Table 4:** Multiple comparison plot: content of virtual backgrounds on VF.

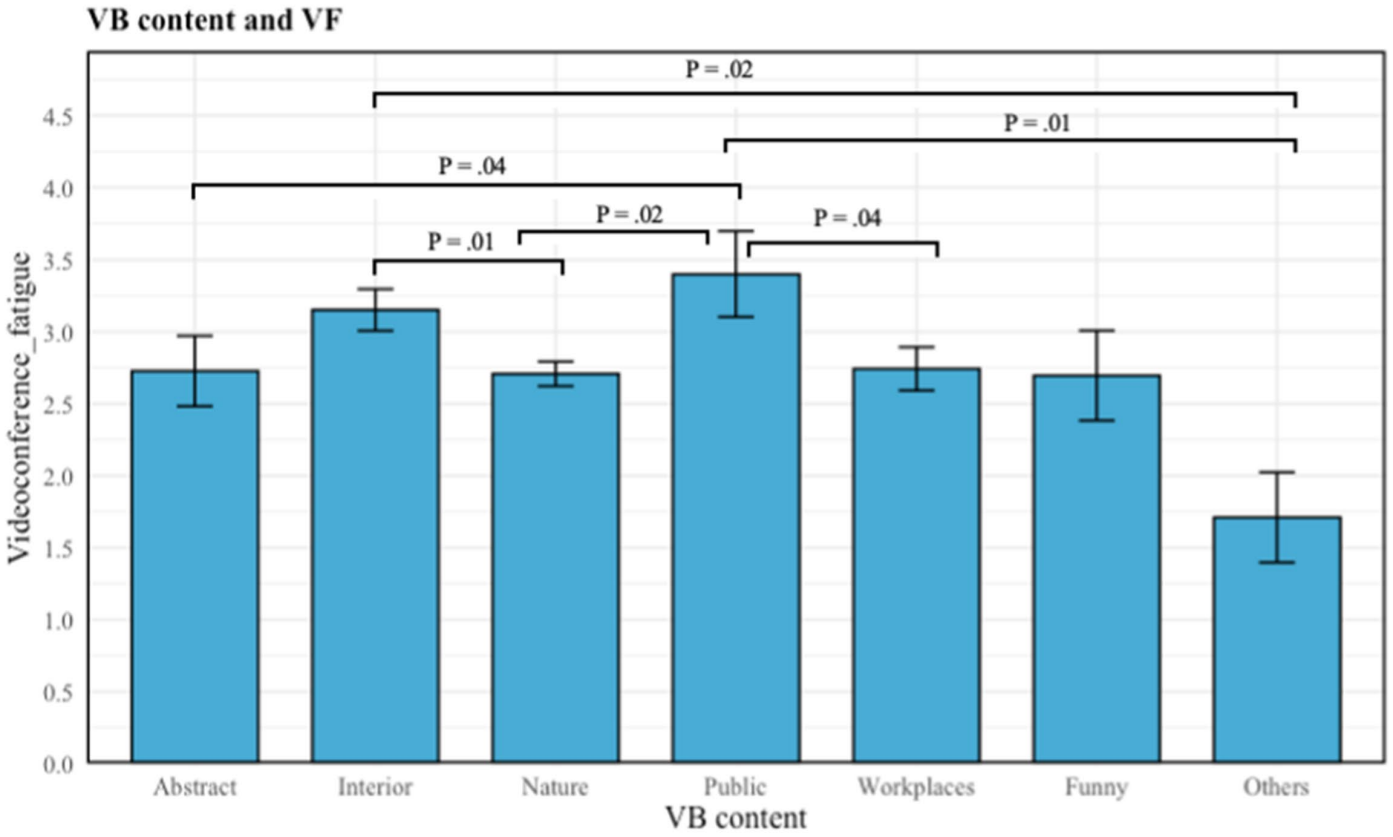

There was a significant difference in VF when using “others” compared to interior spaces (*p* = 0.02) and public spaces (*p* = 0.01) content of virtual backgrounds, with the former experiencing the least VF. In addition, there are significant distinctions in VF between the use of public spaces and abstract (*p* = 0.04), public spaces and workplaces (*p* = 0.04), and public spaces and nature (*p* = 0.02). Participants who chose public spaces experienced higher VF than abstract, workplace, and nature-based virtual backgrounds. Finally, there was also a significant difference in comparison between nature and interior space (*p* = 0.01), with participants using nature-based virtual backgrounds reporting lower VF. Although funny and other had lower average scores than nature, these categories had a much smaller sample size, and the differences were not statistically significant. On the whole, users of nature-based backgrounds reported significantly lower VF compared to those using interior spaces and public spaces as their virtual backgrounds. Hence, we found partial support for H5. In Table 4, in order to view the results more clearly, we marked groups that were significantly different from each other, and did not indicate the non-significant differences.

For H3, the Kruskal-Wallis Test was conducted to examine the differences in cognitive load according to the different types of the virtual background. Significant differences were found among the three categories of type (image, blurred, and video) (*X^2^* (*df* = 2, *p* = 0.05) = 2.96). In this study, the mean value of cognitive load based on different type virtual backgrounds differs accordingly. The mean value of cognitive load in each of the categories are shown in Table 5. Multiple comparisons revealed that the mean value of cognitive load differed significantly between some groups. There was a significant difference in VF when using video compared to blurred (*p* = 0.04) type of virtual backgrounds, with the former experiencing the higher cognitive load (see Table 5).

**Table 5:** Multiple comparison plot: type of virtual backgrounds on cognitive load.

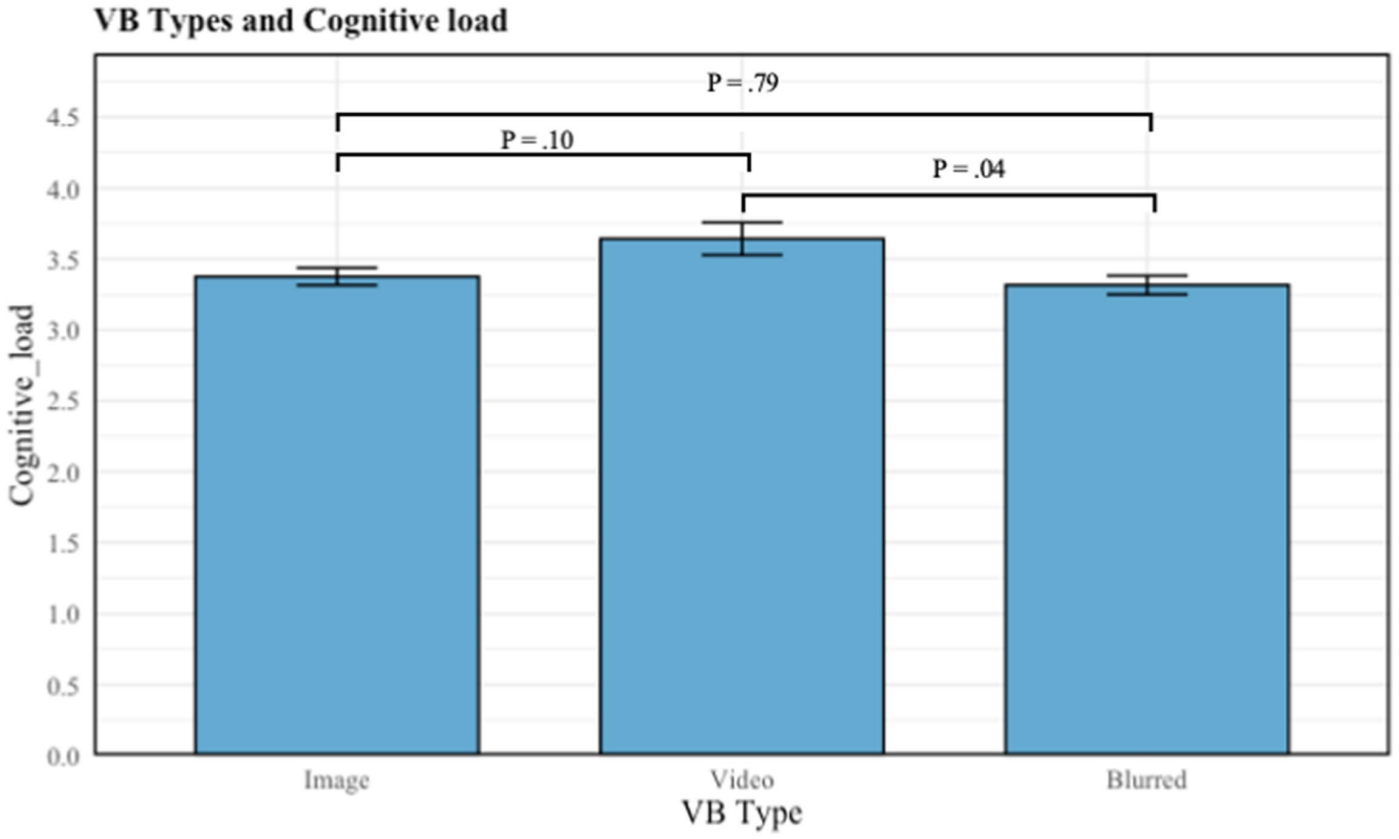

### Mediation model analysis

To examine hypotheses H4a,b,c, PROCESS macro (Hayes, 2013) was used to test mediation model three times, in which image background, video background, and blurred background were entered as a predictor variable, VF was entered as a dependent variable, and cognitive load was entered as the mediator. Age, sex, education, and ethnicity of participants were used as covariates in this analysis. Consistent with previous results, among the four covariates—age, sex, education, and ethnicity—only age has an impact on VF in these three models, while the others were not significant predictors. The results indicate that the older the users, the lower their reported levels of VF. To make the results clearer, these covariates will not be discussed further in the following discussion.

**Figure 1:**
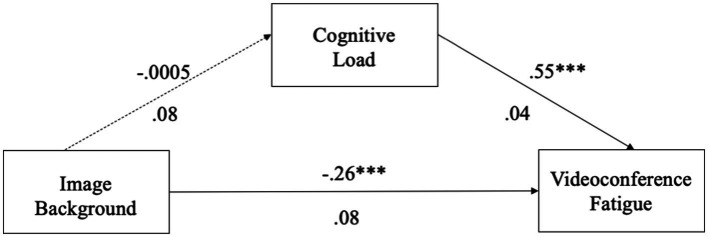
Image background mediation model. *p < 0.05; **p < 0.01; ***p < 0.001.

For the image virtual background model, we found that the use of image virtual backgrounds was not related to cognitive load (*β* = −0.0005, *p* = 0.995, 95% CI = [−0.1698, 0.1688]). Cognitive load was significantly related to VF (*β* = 0.55, *p* < 0.001, 95% CI = [0.4643, 0.6348]). The total direct effect of the use of image backgrounds on VF was significant (*β* = −0.26, *p* < 0.001, 95% CI = [−0.2900, −0.0149]). The bias-corrected 95% CI for the indirect effect via cognitive load (*β* = −0.0003, 95% CI = [−0.0951, 0.0864]) contained zero, indicating a non-significant mediation (see Figure 1). Thus, H4a was not supported.

**Figure 2:**
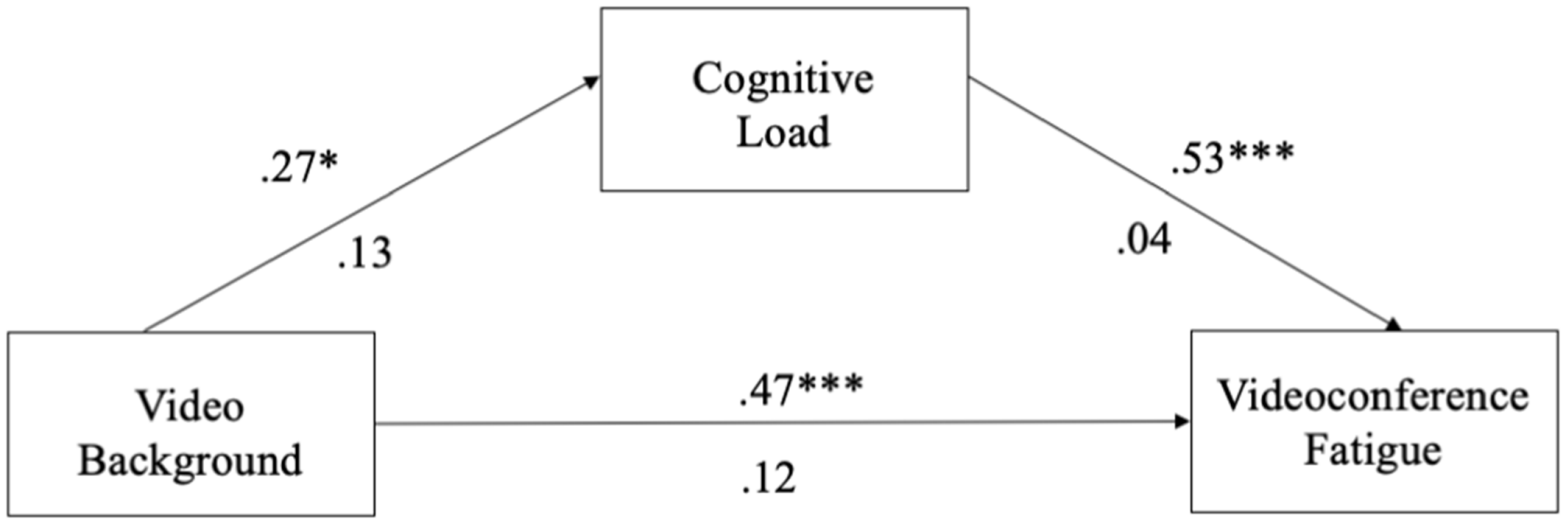
Video background mediation model. *p < 0.05; **p < 0.01; ***p < 0.001.

For the video virtual background model, we found that the use of video virtual backgrounds significantly predicted cognitive load (*β* = 0.27, *p* = 0.036, 95% CI = [0.0175, 0.5214]). Cognitive load was significantly related to VF (*β* = 0.53, *p* < 0.001, 95% CI = [0.4469, 0.6173]). The total direct effect of the use of video backgrounds on VF was significant (*β* = 0.47, *p* < 0.001, 95% CI = [0.2420, 0.7007]). The bias-corrected 95% CI for the indirect effect via cognitive load (*β* = 0.14, 95% CI = [0.0076, 0.2812]) did not contain zero, indicating a significant mediation (see Figure 2). Thus, H4b was supported.

**Figure 3:**
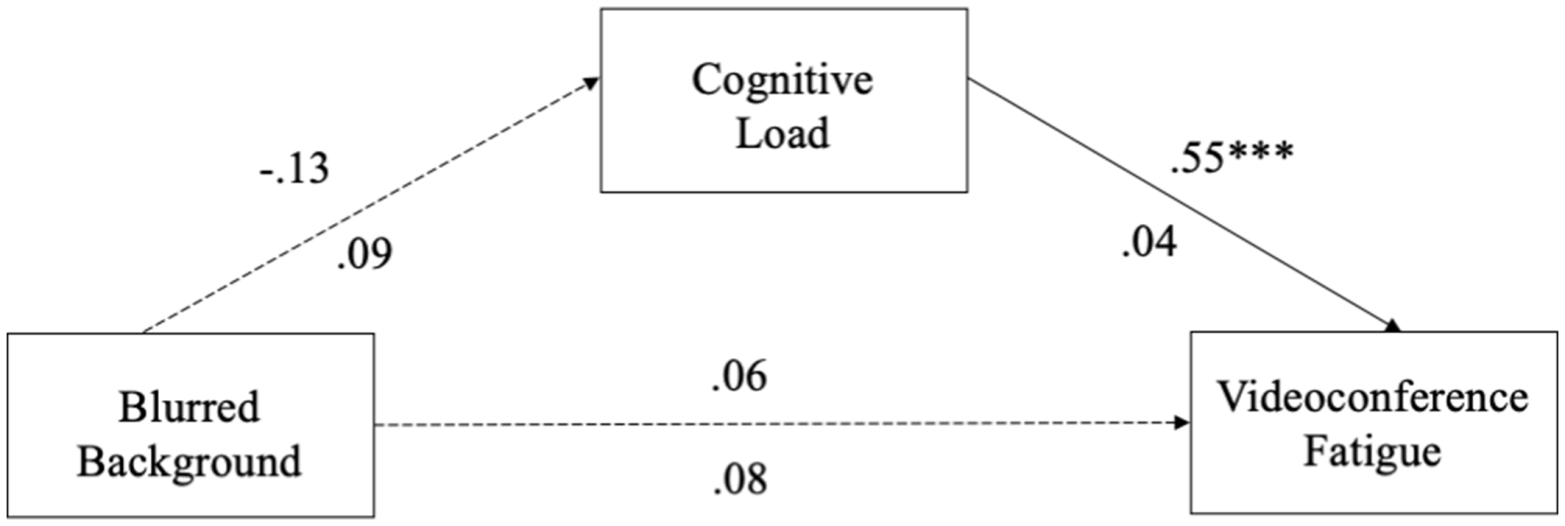
Blurred background mediation model. *p < 0.05; **p < 0.01; ***p < 0.001.

For the blurred virtual background model, we found that the use of blurred virtual backgrounds was not related to cognitive load (*β* = −0.13, *p* = 0.09, 95% CI = [−0.3017, 0.0466]). Cognitive load significantly predicted VF (*β* = 0.55, *p* < 0.001, 95% CI = [0.4652, 0.6381]). The total direct effect of the use of blurred backgrounds on VF was not significant (*β* = 0.06, *p* = 0.49, 95% CI = [−0.1046, 0.2165]). The bias-corrected 95% CI for the indirect effect via cognitive load (*β* = −0.07, 95% CI = [−0.1613, 0.0227]) contained zero, indicating a non-significant mediation (see Figure 3). Thus, H4c was not supported.

## Discussion

With the broadening of videoconference research, an increasing number of studies have indicated that VF can negatively impact users’ psychological and physical well-being (Deniz et al., 2022; Queiroz et al., 2023). In this study, we explored the relationships between virtual background use and VF, in an attempt to understand potential ways to alleviate the negative impact of VF. We discuss our findings below.

First, we found that over 72% of users apply virtual backgrounds during videoconferencing. Image is the most common format of virtual backgrounds applied by these individuals. This result is consistent with the current research on virtual backgrounds that focuses on image, indicating the importance and widespread use of this form of virtual background (Hwang et al., 2021; Palanica and Fossat, 2022; Cook et al., 2023). In addition, the image content of the virtual background can be changed according to the user’s preferences. The majority of users will select a virtual background based on nature images, followed by interior spaces and workplaces, with a minority opting for abstract, public spaces, funny, and other backgrounds.

Our findings revealed that the use of virtual backgrounds cannot help users reduce VF as compared to those who did not use virtual backgrounds. We propose a few reasons for this. First, there are multiple causes of VF, and virtual background use can only account for a partial reduction in the experience of VF. Since the results have shown that using different types of virtual backgrounds result in different levels of VF, the use of virtual backgrounds during videoconferencing there has the potential to alleviate some aspects of VF. Furthermore, selecting inappropriate virtual backgrounds may intensify the experience of VF for users, this was also demonstrated in H5.

The results of H2 indicate that the type of virtual background may lead users who use virtual backgrounds to experience different levels of VF. In terms of virtual background type, for instance, those who use video virtual backgrounds report the highest VF. This aligns with the propositions of LC4MP. When a video is used as the virtual background, people will be directly exposed to new information during the videoconference, which will stimulate OR and lead to the depletion of cognitive resources (Lang, 2017; Fisher et al., 2018). Moreover, a video has the ability to continually provide new information with multiple frames, while the image background is a single frame and will not provide new information. On the other hand, users who choose a blurred background also experience higher VF compared to those who choose an image background. This may be because choosing to use a blurred background can lead to negative emotions among users. Previous studies have demonstrated that using a grey background, similar to the blurred effect, can lead individuals negative emotions and previous research has shown that negative emotions can lead to increased VF (Hwang et al., 2021; Cook et al., 2023; Li and Yee, 2023, p. 183).

The results from H3 and H4a, b, c indicate that there is no significant relationship between image and blurred virtual backgrounds and cognitive load for users who choose to use virtual background. Therefore, cognitive load does not mediate the relationship between image backgrounds and VF, nor between blurred backgrounds and VF. This is because neither image nor blurred backgrounds introduce new information during videoconferencing. Consequently, users of these two types of virtual backgrounds do not receive new information. However, users of video virtual backgrounds experience more cognitive load compared to users of image and blurred backgrounds. This is because video backgrounds are constantly changing, continuously presenting new information to users, consuming cognitive resources, and increasing cognitive load. Therefore, for users of video backgrounds, cognitive load mediates the relationship between video backgrounds and VF.

Finally, based on the results of H5, we found that participants who used public or interior space backgrounds during videoconferencing reported higher levels of fatigue. Previous research on virtual backgrounds has indicated that virtual environments can simulate real-world settings, producing similar effects on users (Palanica and Fossat, 2022). This explains why people using public or interior space backgrounds subconsciously create expectations for themselves to adhere to social norms (Goffman, 1959). According to IMT, users may employ public or interior space backgrounds in videoconferencing to demonstrate professionalism and leave a favorable impression on other participants (Goffman, 1959). However, in the context of videoconferencing, this self-presentation effort can be magnified, leading to increased fatigue. Firstly, when selecting a background, users may worry whether their choice is professional enough, and this concern alone can elevate stress levels (Williams, 2021). Additionally, the pressure to maintain a professional background, even while at home, exacerbates anxiety and fatigue (Shockley et al., 2021). This dual demand increases stress levels, leading to higher VF levels. Increased stress and VF levels subsequently result in decreased well-being.

Additionally, results show that users of “others” and “funny” backgrounds reported lower VF than those using nature-based backgrounds. This may be because these forms of background content are not used for impression management. Individuals who use them may care very little about how these backgrounds portray them, or use these backgrounds simply as a way to conceal their actual physical environment. The resultant lack of stress associated with managing their image leads to lower VF.

### Theoretical and practical implications

From a theoretical perspective, this study expands the scope of the LC4MP and IMT by applying it in understanding the effects of virtual backgrounds on the experience of VF. Previous research on LC4MP has focused on examining the persuasive power and impact on attitudes of specific types of information, particularly text or images (Fisher et al., 2018; Huskey et al., 2020). However, this study explores how virtual environments can also serve as information that influences VF. The findings demonstrate that using different types of virtual backgrounds may affect users’ cognitive load and VF levels. Simultaneously, this study utilizes IMT to explain that people might use various virtual background content to present themselves, but different virtual backgrounds may lead to varying levels of VF experienced by users. Therefore, this research provides a foundation for future studies to explore the role of virtual backgrounds in videoconferencing, particularly in other contexts such as education and the workplace.

From a practical application perspective, we recommend that users who use virtual backgrounds opt for images or blurred virtual backgrounds instead of video backgrounds. Image and blurred virtual backgrounds not only provide less new information to users but also help prevent the introduction of new information during videoconferencing that can occur when no virtual background is used. Additionally, for users who choose image virtual backgrounds, we suggest selecting nature-based backgrounds, as this might be more beneficial for their work and may result in less VF.

### Limitations and future studies

The research is not without limitations. First, because video background was not manipulated, there is no way of knowing if video background causes VF, hence in future research, video backgrounds should be standardized to better understand whether and how they affect VF.

Second, when videoconferencing, users can see not only their own virtual backgrounds but also the virtual backgrounds of other participants. The virtual backgrounds of other attendees can also affect the user. Therefore, future research should investigate whether the virtual backgrounds of other participants have the same impact on the user.

Third, it is entirely possible that some videoconference participants turn off their cameras during videoconferencing or are not in front of their screens all the time, therefore not focusing on the screen and hence rendering their virtual backgrounds irrelevant to their videoconferencing experience. Future studies can consider measuring how often participants are in front of their screens during videoconferencing, or use eye tracking devices to ascertain where participants focus their attention, to account for these possible scenarios.

Also, data in this paper was collected during a single self-report session. Hence, this could raise concerns regarding common method bias. However, as past studies in CMC have often employed this study design (e.g., Oh et al., 2016; Kashian et al., 2017; Antheunis and Schouten, 2020), we feel the exploratory nature of our work makes this a suitable data collection method. Future studies can consider alternative study designs, such as measuring the dependent variables sometime after the study has ended, or use behavioral measures in place of self-report scales, to allay potential concerns.

In this study, participants were asked to think about their general videoconference experiences in answering the questions. However, videoconferencing exists in a variety of contexts, such as in more professional settings like work meetings or school project discussions, or more casual settings such as connecting with friends and family. This may also be a plausible explanation for the age affect seen here, where older users appear to have lower VF than their younger counterparts. Younger videoconference users may be using it for to meet project deadlines, while older adults may be videoconferencing in more casual or relaxed settings. Future research can consider how the use of virtual backgrounds in different contexts may result in VF, especially when impression management might play a stronger role in some contexts (e.g., work) as compared to others.

Moreover, since we did not ask respondents about their specific videoconferencing experiences or personality traits, it is possible that factors external to videoconferencing could have influenced the results. For instance, a person with social anxiety may likely use virtual backgrounds to divert others’ attention away from their face. Their anxiety may also cause them to feel overwhelmed by videoconferencing, regardless of the factors we purposed to explore in this study. Therefore, the relationship between virtual background usage and VF could be attributed to a third factor (in this example, social anxiety). Hence, future research should consider the impact of additional factors, such as social influences and individual differences, on the experience of VF.

Finally, this study did not gather usage information such as participants’ main videoconference platform of choice, videoconference purpose and number of participants on videoconferences, which are all possible factors that can influence the effect of virtual background use on VF. For example, the use of other participants’ virtual backgrounds may influence users’ own experience of VF with the additional information, and the complexity of this relationship may be exacerbated by a large number of participants who all use different virtual backgrounds. Future studies can incorporate these measures and explore how these usage and communication factors might influence virtual background use and reports of VF.

In conclusion, our study explored the impact of virtual backgrounds on user’s well-being in videoconferencing, specifically in the experience of VF. Findings showed that among users of virtual backgrounds during videoconferencing, the type of virtual backgrounds used may have a relationship with the levels of VF experienced by videoconference users. This study offers several recommendations on the use of virtual backgrounds, aiming to assist users in better utilizing the affordances in videoconferencing tools, with the hope of ultimately improving videoconference users’ physical and psychological well-being.

## Data availability statement

The raw data supporting the conclusions of this article will be made available by the authors, without undue reservation.

## Ethics statement

The studies involving humans were approved by the NTU Institutional Review Board. The studies were conducted in accordance with the local legislation and institutional requirements. The participants provided their written informed consent to participate in this study.

## Author contributions

BL: Validation, Project administration, Formal analysis, Data curation, Writing – review & editing, Writing – original draft, Supervision, Resources, Methodology, Investigation, Funding acquisition, Conceptualization. HZ: Writing – review & editing, Writing – original draft, Methodology, Investigation, Formal analysis, Data curation, Conceptualization.
